# Intermittent Scanning Glucose Monitoring or Predicted Low Suspend Pump Treatment: Does It Impact Time in Glucose Target and Treatment Preference? The QUEST Randomized Crossover Study

**DOI:** 10.3389/fendo.2022.870916

**Published:** 2022-05-31

**Authors:** Ulrike Schierloh, Gloria A. Aguayo, Anna Schritz, Muriel Fichelle, Cindy De Melo Dias, Michel T. Vaillant, Ohad Cohen, Inge Gies, Carine de Beaufort

**Affiliations:** ^1^ Department of Pediatric Diabetes and Endocrinology, Clinique Pédiatrique, Centre Hospitalier, Luxembourg City, Luxembourg; ^2^ Deep Digital Phenotyping Research Unit, Department of Precision Health, Luxembourg Institute of Health, Strassen, Luxembourg; ^3^ Competence Center for Methodology and Statistics, Luxembourg Institute of Health, Strassen, Luxembourg; ^4^ Institute of Endocrinology, Sheba Medical Center, Tel Hashomer, Israel; ^5^ Pediatric Endocrinology, KidZ Health Castle, UZ Brussel, Vrije Universiteit Brussel, Brussels, Belgium

**Keywords:** children, type 1 diabetes, insulin pump, iscCGM, predicted low suspend function

## Abstract

**Objective:**

To compare glycemic control and treatment preference in children with type 1 diabetes (T1D) using sensor augmented pump (SAP) with predictive low glucose suspend (SmartGuard^®^) or pump with independent intermittent scanning continuous glucose monitoring (iscCGM, Freestyle libre ^®^).

**Methods:**

In this open label, cross-over study, children 6 to 14 years of age, treated with insulin pump for at least 6 months, were randomized to insulin pump and iscCGM (**A**) or SAP with SmartGuard^®^ (**B**) for 5 weeks followed by 5 additional weeks. The difference in percentages of time in glucose target (TIT), (3.9 – 8.0 mmol/l), <3 mmol/l, > 8 and 10 mmol/l, were analyzed using linear mixed models during the final week of each arm and were measured by blinded CGM (IPro2^®^).

**Results:**

31 children (15 girls) finished the study. With sensor compliance > 60%, no difference in TIT was found, TIT: **A** 37.86%; 95% CI [33.21; 42.51]; **B** 37.20%; 95% CI [32.59; 41.82]; < 3 mmol/l **A** 2.27% 95% CI [0.71; 3.84] **B** 1.42% 95% CI [-0.13; 2.97]; > 8 mmol/l **A** 0.60% 95% CI [0.56, 0.67]; **B** 0.63% [0.56; 0.70]. One year after the study all participants were on CGM compared to 80.7% prior to the study, with a shift of 13/25 participants from iscCGM to SAP.

**Conclusions:**

In this study, no significant difference in glycemic control was found whether treated with SAP (SmartGuard^®^) or pump with iscCGM. The decision of all families to continue with CGM after the study suggests a positive impact, with preference for SmartGuard^®^.

**Clinical Trial Registration:**

[clinicaltrials.gov], identifier NCT03103867.

## Introduction

To prevent short-and long-term complications, patients with type 1 diabetes (T1D) need an optimal metabolic control (1), which is challenging, especially for children (2).

Augmenting the insulin pump with glucose sensor information has shown to improve outcome (3). While continuous glucose monitoring is associated with decreased HbA1c levels and reduced time spent in hypoglycemia in individuals with T1D using insulin pump therapy in long-term studies, better outcomes depend on longer and continuous sensor use (3).

The use of technologies like sensor augmented insulin pumps and hybrid closed loop systems is increasing in children and adolescents with diabetes. These devices are not globally accessible, whereas continuous glucose measurements (CGM) has become increasingly available.

Minimed 640G^®^ pump with SmartGuard^®^ function combines alerts with an automated basal insulin suspension for prediction of low glucose, in order to prevent a hypoglycemia event. Alerts can be set on or off but the low threshold alert is mandatory (4).

A multicenter study in pediatric diabetes patients showed that SmartGuard^®^ technology showed a significant reduction in risk of hypoglycaemia without increasing HbA1c (5).

Freestyle Libre**^®^** is another device measuring continuously the interstitial glucose levels. Results can be obtained when the patient/caregiver actively scans the sensor (iscCGM). No alerts are given when glucose values increase or decrease and no communication exists between the glucose measurement and the insulin pump (4).

The evaluation of iscCGM being as safe as self-monitoring of blood glucose (SMBG) and resulting in a better metabolic outcome than SMBG was demonstrated in children (6, 7).

The impact of these two technologies on metabolic control has been studied previously (8). The sensor augmented pump offers real time glucose values and alerts in case of hypo-and hyperglycemia and a predicted low glucose suspend of insulin infusion. However, concerns of alarm fatigue have been raised (9), though no data on Minimed 640G have been published. An alternative might be the intermittent glucose scanning to obtain glucose values when desired on the persons own initiative. We designed this study in order to get more information about the impact of the technology on metabolic control. Furthermore, we evaluated what device the families choose based on experience with both technologies after finishing the study. We are not aware of any study comparing these two technologies in children.

The objective of this study was to evaluate the impact on time in glucose target (TIT), 3.9 – 8.0 mmol/l, in children with T1D, comparing a sensor augmented insulin pump (Minimed 640G^®^ with SmartGuard^®^ technology) to the use of the same insulin pump with an intermittent scanning continuous glucose monitoring device (iscCGM; Freestyle libre^®^) that does not interact with the pump.

## Methods

This trial was registered (ClinicalTrials.gov, NCT03103867) and details of the methodology are described elsewhere (4).

### Study Design

The study had an open-label, single -center, randomized, two-period crossover design.

Ethical approval from the Luxembourgish National Ethics Commission for the final study was obtained before the start of the study.

In our center we take care of more than 350 patients with diabetes, 90.4% with type 1 diabetes. In this group 85% use a CGM and 62% are pump–users (SWEET report, March 2022). All our patients and their caregivers regularly undergo an age specific diabetes and nutritional educational program at diagnosis and afterwards in our outpatient survey.

### Participants

We included participants that fulfilled the following inclusion criteria: between 6 and 14 years of age, T1D for at least 6 months, on insulin pump treatment for at least 6 months, and HbA1c ≤ 11% (≤ 96.72 mmol/mol). These were patients from the pediatric diabetes consultation at the Children’s Hospital in Luxemburg.

Exclusion criteria were physical or psychological disease likely to interfere with an appropriate conduct of the study. Prior to enrolment, written informed consent was obtained from the parents and all children gave their informed assent.

### Sample Size

A sample size of 36 patients with a minimum of 31 patients was calculated for a power of 80% (4).

### Randomization

Randomization (ratio 1:1) was performed by a statistician with 4 blocks of 8 sequences and treatment allocation based on prepared envelopes with the sequence code (**A-B** or **B-A**). After consenting, the envelope was opened by the medical team to provide the participant with the allocated treatment sequence (4). Blinding was not possible for the participant nor the medical team.

### Procedures

After signing the informed consent/assent, subjects were randomized either to **treatment A**, insulin pump Minimed ^®^ 640G and independent interstitial glucose measurement (Freestyle libre^®^) or to **treatment B**, SAP with the SmartGuard feature (Minimed^®^ 640G), each for 5 weeks. Following a 3 week washout period, subjects crossed over to the other study arm for another 5 weeks. Further details are available and published elsewhere (4).

Freestyle libre^®^, which was used in our study, has no alarms to alert when high or low glucose values are measured. This has changed in the more recent variant, the Freestyle libre 2^®^, where an alarm option has been included.

Study visits occurred at randomization (baseline), at treatment start (V1-start first allocation, V3-start second allocation) and at the end (V2-end first allocation, V4-end second allocation) of each treatment period. There were no study visits during the washout period.

Demographic variables were collected at baseline. HbA1c measurements (DCA Vantage^®^, Siemens) were performed at each visit (V1-V4). A blinded CGM (I-Pro2^®^) was used to evaluate TIT during the last week of each treatment arm.

The use of the two glucose measurement tools and the features of the Minimed ^®^640G pump were explained during the training visit V1. All participants had access to a 24/7 diabetes hotline in case of technical or any other issues.

Settings of the Smart Guard were standardized based on the current experience (10). The low limit was set at 3.4 mmol/l, with an insulin suspension at ≤7.3 mmol/l if the predicted value within 30 minutes was 4.5 mmol/l. Parent/patient were informed before insulin was suspended by an alert (4).. At V2, I-Pro2^®^ was placed for 7 days and the patient received instructions to perform two glucose measurements per day for calibration. After that week, the device was collected for analysis.

Thereafter, during the 3 week washout period, the 640G pump was maintained but in combination with a minimum of four blood glucose measurements and no iscCGM nor rtCGM. After the washout period, the second treatment period started with visit V3, on either Freestyle Libre^®^ or SmartGuard^®^.

At visit V4, after 4 weeks of the second treatment arm, the I-Pro2^®^ was placed again with the same request to perform 2 blood glucose values per day during 7 days. After this week, all devices were collected for analysis and the patient restarted his/her usual pre-study treatment.

During the 7 days-period with blinded CGM, the patients continued their assigned treatment (SAP or iscCGM and insulin pump).

**The primary endpoint** was defined as the percent of time spent in glucose target, TIT, (3.9 - 8 mmol/l) of **treatment A** and **B** during the final 7 days of a five-week device arm. This was measured by a blinded CGM (IPRO2^®^) at week 5 (V2) and 13 (V4), for participants with glucose sensor compliance > 60% during this week. As published in our protocol (4), we set out intending to use 6 days for analysis. After completion of the study and taking into account that we had enough data, we decided to analyze 7 days instead of 6.

**Secondary endpoints** included the between arm difference in percentage of time spent below glucose target (< 3.0 mmol/l) and above glucose target (> 8 and >10 mmol/l.

Severe hypoglycemic events (definition according to the current ISPAD guidelines (11) were documented.

### Data Management and Data Quality

TIT and between arm differences were evaluated by the blinded CGM (IPro2^®^). Details on the data extraction have been summarized previously (4). Data from the blinded CGM were extracted by Medtronic GlyVaRT software tool, and pump data were transferred through Contour Next Link^®^ glucose meter to Medtronic CareLink therapy Management Software. Data quality was ensured in the data management process by double entry using Ennov Clinical and including online logical controls to detect outliers and missing information. Freestyle Libre^®^ or SmartGuard^®^ data were only used by the patients for daily treatment adjustments.

### Statistical Analysis

Baseline characteristics were described using mean [standard deviation (SD)], median [25% quartile (Q1), 75% quartile (Q3)] for normal and not normal distributed continuous variables, respectively, and frequencies (percentages) for binary and categorical variables.

#### Baseline Characteristics for Children

Age (years), sex, height (cm), weight (kg), BMI (kg/m^2^ and z-scores), Hba1c (%, mmol/mol) duration of diabetes, duration of pump use (years), glucose value, percent of time within, below or above defined glucose target.

#### Primary Outcome

The percentage of time spent in glucose target, TIT, (3.9 - 8 mmol/l) of treatment A and B was analyzed by using a linear mixed model with treatment, sequence of treatments, and period as fixed effects and patient as random intercept effect.

#### Secondary Outcome

Below glucose target (< 3.0 mmol/l) and above glucose target (> 8.0 mmol/l and > 10.0 mmol/l) during the final 7 days of a 5 week device arm measured by blinded CGM during week 5 and week 13 were compared between device arms using a linear mixed model with device, sequence and period as fixed effects and patients as random effect. Only data with a sensor compliance of > 60% were included in the analysis.

#### Safety Outcome

The number of severe hypoglycemic events in both treatment arms, defined by ISPAD (11), was analyzed through a table of frequencies.

We performed a sensitivity analysis including all patients in the analysis regardless of their compliance.

## Results

**Figure 1:**
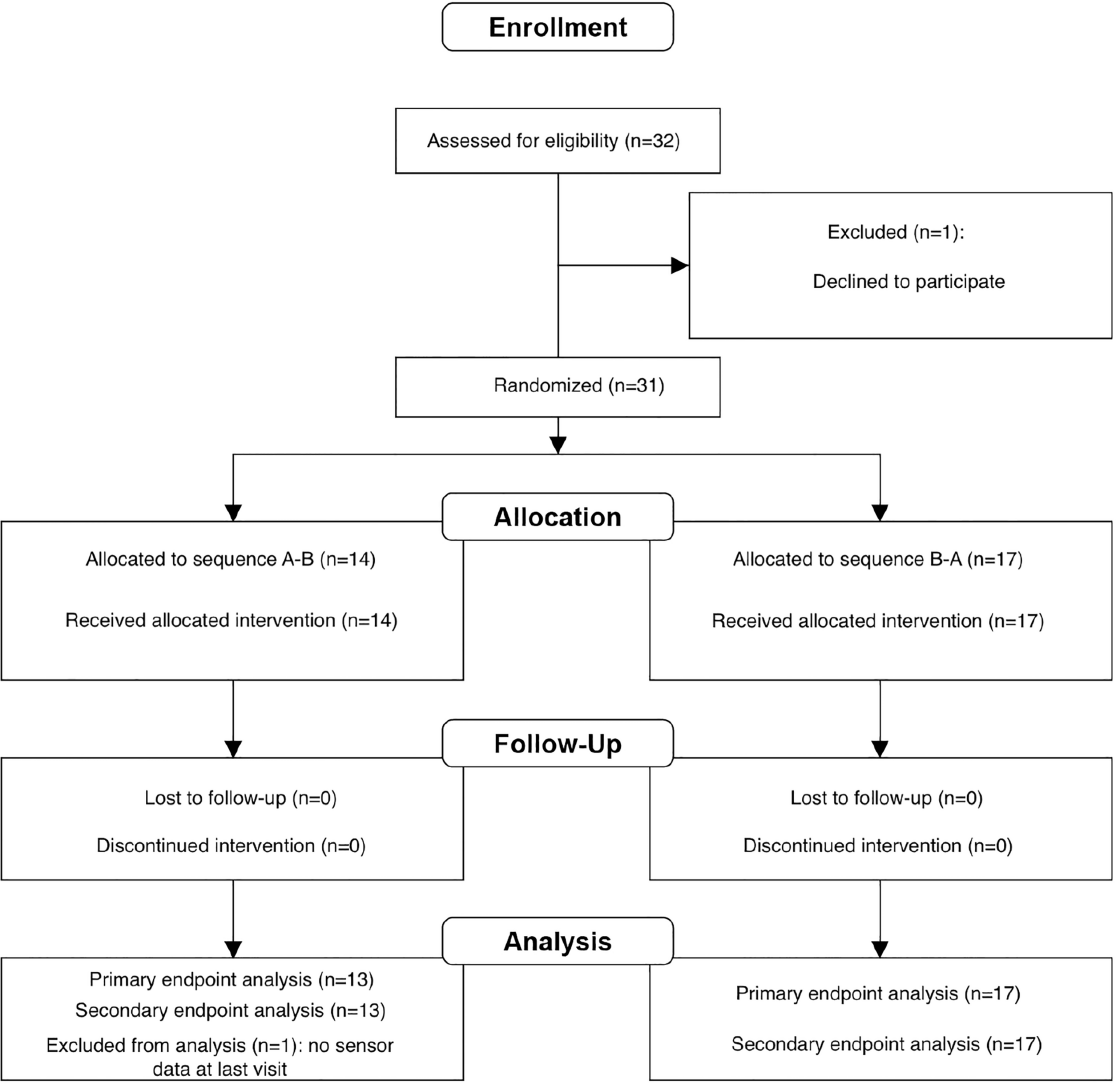
Subject disposition.

A total of 32 children (15 girls), 6 to 14 years of age, mean HbA1c 7.5% (SD 0.6), 58.1 mmol/mol, (SD 6.5) and mean diabetes duration of 5.9 years (SD 3.29), and on insulin pumps consented to participate in this study. Prior to the study, 25 subjects used CGM (iscCGM). As one child dropped out of the study before randomization, based on non-compliance with the use of the sensor, these data were not included. **Figure 1** shows the subject disposition.

For one participant the glucose sensor values of the last visit were missing, therefore 30 participants were analyzed for primary and secondary endpoints. **Table 1** shows the demographic baseline values for study participants (31 children).

**Table 1:** Descriptive baseline characteristics of the participating children.

	All	Sequence A-B	Sequence B-A
N	31	14	17
Age, years	10.5 (2.3)	10.8 (2.0)	10.2 (2.5)
Female, N (%)	15 (48.4)	7 (50.0)	8 (47.1)
Caucasian, N (%)	30 (96.8)	13 (92.9)	17 (100)
Height, cm	143.7 (14.6)	145.6 (15.2)	142.1 (14.4)
Weight, kg	42.8 (13.2)	44.1 (15.7)	41.8 (11.2)
BMI, kg/m^2^	20.2 (3.1)	20.1 (3.9)	20.3 (2.5)
Z score BMI^a,b^	1.0 (0.4, 1.3)	1.0 (0.5, 1.2)	1.1 (0.5, 1.3)
HbA1c, %	7.5 (0.6)	7.6 (0.6)	7.3 (0.5)
HbA1c, mmol/mol	58.1 (6.5)	59.9 (6.9)	56.6 (5.9)
Diabetes duration, years^b^	5.6 (3.0, 8.1)	5.7 (3.7, 7.1)	5.6(2.9, 9.8)
Pump use, years^b^	4.0 (2.1, 5.1)	3.9 (2.4, 6.9)	4.5 (1.8, 9.1)

Data are mean (SD), median (Q1, Q3) or n (%).

a Z scores BMI are calculated with the formula z-score = (X-m)/SD; X=BMI; m=mean, SD=standard deviation of BMI of the reference population with same sex and age.

b Median (Q1, Q3) (variables with non-normal distribution).

### Primary Endpoint

#### Percentage of Time in Glucose Target (3.9 – 8.0 mmol/l)

Only data with a sensor compliance of > 60% were included in the analysis (**Table 2**).

**Table 2:** Linear mixed model of time in glucose target.

	Coefficients (95% confidence intervals)
Intercept	36.4 (30.0; 42.9)*
Time to target device B	-0.65 (-6.2; 4.7)
Period (visit V4)	0.09 (-5.3; 5.55)
Sequence of device Arm B-A	2.7 (-4.5, 9.8)

*P <0.05.

We analyzed the data of sensor compliance > 70%. As there was no statistically significant difference, we show the sensor compliance > 60%, in order to include a maximum of patients.

**Figure 2:**
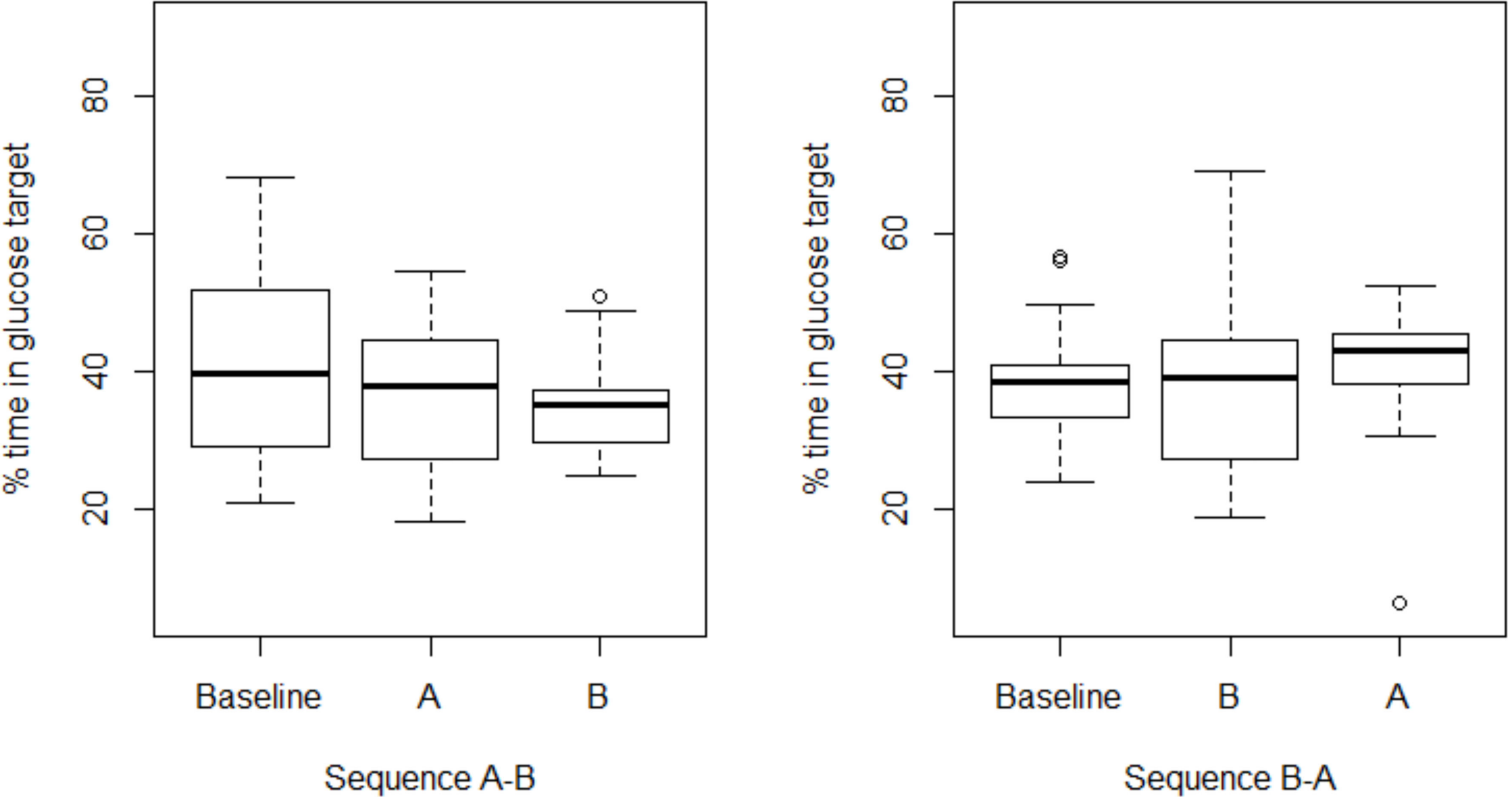
Boxplot of percentage in glucose target, sensor compliance > 60%.

Treatment **A** had an adjusted mean percentage in glucose target of 37.86% [95% CI (33.21; 42.51)]. The adjusted mean percentage in glucose target in treatment **B** was 37.20% [95% CI (32.59; 41.82)]. No significant difference between treatment **A** and **B** was found (p-value = 0.817). No carry-over effect was observed (2.74; SE =3.66; p-value = 0.461). Interestingly, we observed in both sequences a reduced variability in TIT in the second treatment arms (**Figure 2**).

In a sensitivity analysis including all patients in the analysis (ignoring the compliance of the glucose sensor) no statistically significant difference could be found (data not shown).

### Secondary Endpoints

#### Severe Hypoglycemia

No severe hypoglycemia occurred in both treatment arms.

#### Percent Time of Glucose < 3.0 mmol/l

No significant differences between treatment **A,** insulin pump and iscCGM, [2.27% 95% CI (0.71; 3.84)] and **B**, SAP with SmartGuard^®^ function, [1.42% 95% CI (-0.13; 2.97)] could be found for percent time below 3.0 mmol/l.

One child showed a high percent time of glucose < 3.0 mmol/l with 24.91% in treatment A versus 0% in treatment B compared to other children (median = 0.815%). Linear mixed model excluding one observation with high outcome value lowered the least square means (marginal means extracted from the model fitting the data) of percent time < 3.0 mmol/l, but no significant difference between device A and B was found (**Table 3**).

**Table 3:** Glucose time percentage < 3.0 mmol/l: p-value=0.4460.

Least Square Means									
Effect	treatment	Estimate	Standard Error	DF	t Value	Pr > ItI	Alpha	Lower	Upper
Treatment	A	2.2729	0.7772	47	2.92	0.0053	0.05	0.7093	3.8364
Treatment	B	1.4210	0.7707	47	1.84	0.0072	0.05	-0.1294	2.9713

Most children had no low glucose values < 3.0 mmol/l, therefore the outcome variable shows a high number of zero percentages (19 out of 51 observations, 37.25%).

#### Percent Time of Glucose > 8 mmol/l and > 10 mmol/l

No significant association between devices A [0.60%; 95% CI (0.53, 0.67)] and B [**B** 0.63%;95% CI (0.56; 0.70)] and percent time of glucose > 8 mmol/l (p-value: 0.463), **Table 4**, and percent time of glucose > 10 mmol/l was found (p-value: 0.996), **Table 5**.

**Table 4:** Glucose time percentage > 8.0 mmol/l; p-value 0.463.

Least Square Means									
Effect	treatment	Estimate	Standard Error	DF	t Value	Pr > ItI	Alpha	Lower	Upper
treatment	A	0.5971	0.0352	44.0	16.98	<2.2	0.05	0.5262	0.6680
treatment	B	0.6264	0.0349	43.8	17.94	<.2.2	0.05	0.5560	0.6968

**Table 5:** Glucose time percentage > 10.0 mmol/l; p-value 0.9955.

Least Square Means									
Effect	treatment	Estimate	Standard Error	DF	t Value	Pr > ItI	Alpha	Lower	Upper
Treatment	A	38.8971	2.7722	43.4	14.03	<2.2	0.05	33.3077	44.4865
Treatment	B	38.9141	2.7531	43.1	14.13	<2.2	0.05	33.3622	44.4661

#### Treatment Choice 1 Year After Completing the Study

Prior to the study, 6 out of the 31 participants had no regular experience with a continuous glucose measurement, whereas 1 year after the QUEST study, all 6 children used one of the two CGM continuously (2 on iscCGM Freestyle libre^®^ and 4 used the SAP with SmartGuard^®^ function).

One year after the study 14 out of the 25 participants (56%) with prior regular CGM experience had changed their CGM treatment to the sensor augmented pump option with SmartGuard^®^.

Eight out of 11 patients (73%) who stayed on iscCGM after the study chose this option because of no need to calibrate the sensor.

#### Reported Harms

No specific harms were reported.

## Discussion

In this study, with real-life data using 2 different CGM systems, we did not identify any significant difference in TIT, time below and above target comparing the same insulin pump with two different glucose monitoring systems (iscCGM without alerts compared to the SmartGuard^®^ feature with alerts and predicted low glucose suspend). Based on their experience in the study, even those participants without prior regular CGM use decided to continue the CGM after the end of the study. The use of the CGM itself, iscCGM or rtCGM, seems most relevant for the outcome. This is supported by other studies, exploring participants’ experiences (12) or treatment adjustments based on sensor information, iscCGM or rtCGM (13).

Although no carry over effect was observed, the reduced variability in TIT suggests that CGM use over time influences diabetes control. The continuous information on glucose levels allows a faster insulin adjustment. These targets may differ between the different participants and different treatments modalities. Hypoglycemia fear, alarm fatigue, family interactions, and more or less confidence in devices or diabetes distress may influence individual target setting and the choice and use of CGM. A recently published review on psychological outcomes in children using iscCGM or rtCGM clearly suggests to consider these human factors while counseling families in their choice of CGM (14).

A limitation of our study is the relatively short study and analysis duration and the limited number of participants. As the blinded sensor had to be changed after 7 days, the evaluation was limited to 7 days to prevent potential drop outs. We controlled for the small number by the study design. As the data are all obtained in the free living at home, they do reflect the real world situation, which in our observation represents the strength of this study.

Even if recent technological development towards closed loop systems shows further near normalization of metabolic control, many countries do not have access to these technologies. The access to CGM, with or without connection to an insulin pump, increases and remains very important to optimise the outcome (15).

Despite the use of the pumps/sensors, glucose time in target in our and other populations remained insufficient (16). Although fast progress in technology is observed, human factors are important to ensure optimal use and outcome. Reduced burden for the patients and families should be considered (17).

## Data Availability Statement

The raw data supporting the conclusions of this article will be made available by the authors, without undue reservation.

## Ethics Statement

The studies involving human participants were reviewed and approved by Comité National d’Ethique de Recherche, Luxembourg. Written informed consent to participate in this study was provided by the participants’ legal guardian/next of kin.

## Author Contributions

US and CB: concept of the study and design, protocol, recruitment, data analysis and writing of the paper; MF and CM: recruitment and conduct of the study; GA: study design, data management and data analysis; AS: data management and data analysis; IG: concept of the study; MV: concept and design of the study; OC: protocol; All authors read and approved the final manuscript.

## Funding

This is an investigator-initiated study. Medtronic contributed in kind to the devices (insulin pumps, transmitter, I-Pro2®) and to the accessories (glucose sensors).

## Conflict of Interest

The sponsor of this project had the right of commenting but the authors retained the right to accept or reject comments or suggestions.

OC is an employee of Medtronic. CB has received a honorary for giving talks on devices, developed by Medtronic, and has been member of Medtronic EU Psychology Advisory board (the development of an e learning tool on diabetes and adolescence).

The remaining authors declare that the research was conducted in the absence of any commercial or financial relationships that could be construed as a potential conflict of interest.

## Publisher’s Note

All claims expressed in this article are solely those of the authors and do not necessarily represent those of their affiliated organizations, or those of the publisher, the editors and the reviewers. Any product that may be evaluated in this article, or claim that may be made by its manufacturer, is not guaranteed or endorsed by the publisher.
